# Responses of Crop Pests and Natural Enemies to Wildflower Borders Depends on Functional Group

**DOI:** 10.3390/insects8030073

**Published:** 2017-07-25

**Authors:** Ellie McCabe, Gregory Loeb, Heather Grab

**Affiliations:** 1 Department of Biological Sciences, University of New Hampshire, Durham, NH 03824, USA; ellieannmccabe@gmail.com; 2 Department of Entomology, New York State Agricultural Experiment Station, Cornell University, Geneva, NY 14456, USA; gme1@cornell.edu; 3 Department of Entomology, Cornell University, Ithaca, NY 14853, USA

**Keywords:** wildflower planting, pests, natural enemies, functional group

## Abstract

Increased homogeneity of agricultural landscapes in the last century has led to a loss of biodiversity and ecosystem services. However, management practices such as wildflower borders offer supplementary resources to many beneficial arthropods. There is evidence that these borders can increase beneficial arthropod abundance, including natural enemies of many pests. However, this increase in local habitat diversity can also have effects on pest populations, and these effects are not well-studied. In this study, we investigated how wildflower borders affect both natural enemies and pests within an adjacent strawberry crop. Significantly more predators were captured in strawberry plantings with wildflower borders versus plantings without wildflowers, but this effect depended on sampling method. Overall, herbivore populations were lower in plots with a wildflower border; however, responses to wildflower borders varied across specific pest groups. Densities of *Lygus lineolaris* (Tarnished Plant Bug), a generalist pest, increased significantly in plots that had a border, while *Stelidota geminata* (Strawberry Sap Beetle) decreased in strawberry fields with a wildflower border. These results suggest that wildflower borders may support the control of some pest insects; however, if the pest is a generalist and can utilize the resources of the wildflower patch, their populations may increase within the crop.

## 1. Introduction

Complex agricultural landscapes support a diverse community of beneficial insects and ecosystem services that in turn support crop productivity [1,2,3,4]. Yet, for most of the 20th century agricultural landscapes have become increasingly homogeneous due to increased expansion of specialized, monoculture production systems [5]. Loss of diversity and structural complexity from agro-ecosystems is one of the primary drivers of declines in biodiversity and associated ecosystem services worldwide [6,7]. In particular, the loss of habitat diversity in agro-ecosystems leads to a reduction in the abundance and diversity of beneficial insects including pollinators and natural enemies [4,8,9,10]. In recent years, there has been significant interest in practices that increase farmland diversity in ways that restore ecosystem services while maintaining crop productivity [7,11,12]. 

One practice that has been explored in a number of cropping systems is the addition of wildflowers to crop borders. Wildflowers provide resources to natural enemies of crop pests including shelter from disturbance and overwintering habitat as well as a source of nectar, pollen, and alternative prey [13,14]. Wildflower borders have been found to increase predator populations in the crop when planted next to blueberries [15,16], cabbage [17,18], wheat [19,20] and tomato [21]. Nevertheless, wildflower plantings are not always successful at increasing natural enemy populations [22,23,24] due to both local and landscape level effects [25]. Landscape level effects include lack of a source population of natural enemies in the surrounding habitat or that natural habitat surrounding the crops can be too small and/or too far away for natural enemies to colonize the wildflower and crop habitats [25,26,27]. Local effects include farm management practices that can affect the establishment of natural enemies such as the use of broad-spectrum insecticides [28]. 

The addition of wildflower plantings in field margins can also have direct effects on crop pests. Wildflower borders may be a source for pest populations as well as beneficial insects. Pests can use the strips as refuge from disturbances or as overwintering sites. Generalist pests can feed on the flowering plant species throughout the summer [29]. In cases where wildflowers are a better resource for pests than they are for natural enemies, the population of pests in the wildflower border may spill over into the crop and increase crop damage. However, not all pests are expected to benefit from wildflower borders. Specialist pests that do not utilize the wildflowers as a resource for habitat or food may be negatively affected by the increased local plant diversity by interfering with host plant location [30,31]. 

In this study we explore the effect of adding wildflower borders to strawberry (*Fragaria x ananassa*) plantings on natural enemy and pest populations. *Lygus lineolaris* Say (Tarnished Plant Bug; Hemiptera: Miridae) and *Stelidota geminata* Say (Strawberry Sap Beetle; Coleoptera: Nitidulidae) are two of the most economically significant pests of strawberries grown in the Northeastern USA, therefore we have explicitly evaluated their populations separately from other herbivores surveyed. 

The tarnished plant bug, *L. lineolaris*, is a generalist pest known to feed on over 300 species of plants [32]. Without control efforts, it can damage up to two-thirds of a strawberry crop [33]. *L. lineolaris* overwinters in protected areas including leaf litter, hedgerows, or plant debris [34] and higher densities have been observed in blueberry and tomato fields with wildflower borders [16,35]. Alternatively *S. geminata*, a fruit-feeding specialist, does not appear to benefit from wildflower plantings [16]. *S. geminata* has increased as a pest in strawberries due to the operation of pick-your-own strawberry fields that leave ripe strawberries in the field [36]. Despite its common name, *S. geminata* is not a specialist of strawberry; the adult and larva feed on ripe fruit of many different genera including crops such as raspberry, blueberry, apple, melon, and sweet corn [37]. Adults overwinter in wooded areas or in blueberry and raspberry plantings [38]. Predator prey relationships are not well described for either species with the exception of specialist parasitoids [39,40]. The eggs, immatures and adults of both species are not known to be chemically protected and would be an appropriate sized prey for carabids, spiders, huntsman and other generalist arthropod predators 

In this study, we hypothesized that (1) natural enemy abundance will be greater in crop plantings with a wildflower margin compared to control plots without a wildflower border; and (2) the abundance of the generalist pest, *L. lineolaris*, will remain stable or even increase in crop plantings with adjacent wildflower borders, while *S. geminata*, a pest that does not utilize the floral resources of a wildflower strip, will decrease in crop plantings with adjacent wildflower borders. 

## 2. Methods

The study was conducted in the summer of 2014 on six research farms in the area around the New York State Agricultural Experiment Station in Geneva, NY, USA. On each farm, two 10 × 15 m experimental plots consisting of five rows of strawberry (var. “Jewel”) were established in the spring of 2012. Plots were managed without use of fungicides or insecticides and weeded by hand with the exception of a pre-emergent herbicide applied in the fall of 2013. Plots were separated by a minimum of 200 m and were randomly assigned to either a control border or a native perennial wildflower planting. Composition and management of control borders were representative of field edge management practices in the region and consisted primarily of orchard grass (*Dactylis glomerata* L.; Poaceae), which was regularly mown over the growing season. Wildflower borders were established in the fall of 2012 along the edge of one of the outside rows of the strawberry plantings. Wildflower plantings were 4 m wide by 10 m long and consisted of the following 11 US native perennial species: *Zizia aurea* (Apiaceae), *Penstemon digitalis* (Plantaginaceae), *Coreopsis lanceolata* (Asteraceae), *Potentilla fruticosa* (Rosaceae), *Vironicastrum virginicum* (Plantaginaceae), *Agastache nepetoides* (Laminaceae), *Silphium perfoliatum* (Asteraceae), *Lobelia siphilitica* (Campanulaceae), and *Solidago altissima* (Asteraceae). These species were selected based on their attractiveness to beneficial insects [41,42] and provide overlapping bloom periods so that flowers are present throughout the growing season. 

Pest surveys were conducted in each strawberry planting during the fruit ripening period in June 2014. This period was selected because it is the window in which damage caused by these fruit feeding pests occurs. All plots were sampled on the same day. Insects were collected by vacuuming once along all five rows of each planting for approximately 5 minutes with a modified D-VAC type suction sampling device (Echo ES 230 Shred ‘n Vac, Lake Zurich, IL, USA, 20 cm cone diameter). The contents of the sample were placed in an ethyl acetate kill jar before being frozen at −20 °C. Frozen samples were later sorted and known economically important pests including *L. lineolaris* and *S. geminata* were identified to species. All remaining arthropods were identified to order or family and then placed into functional groups based on the predominant life history exhibited by their taxonomic group (i.e., herbivorous or predacious).

Additionally, pitfall traps were deployed between strawberry rows (n = 4) in each plot to better characterize the ground dwelling insect communities. Functionally important predators in the system such as spiders and carabid beetles are more likely to be collected by this method [43,44,45]. Therefore, vacuum and pitfall samples were used as complimentary methods to estimate natural enemy community composition. Pitfall traps consisted of 16 oz SOLO brad cups set flush with the soil surface and filled with 50 mL of a 5% dish soap killing solution. Traps were deployed over a three-day period once in each plot. 

To determine the impact of wildflower strips on the abundance of different functional groups we used generalized linear mixed effect models with a poison error distribution. Response variables included the abundance of each functional group or taxa and fixed effects included the interaction between functional group class and plot treatment (wildflower border or control). Random effects included treatment within farm to account for the nested experimental design. Abundances from vacuum samples and pitfall traps were modeled separately. Pairwise contrasts for the difference between abundances in control and wildflower treatments plots were performed using the pairs function and the lsmeans package in R. Differences in community composition between sampling types and crop border treatments were assessed using permutational-MANOVA with 999 permutations on Bray-Curtis dissimilarities. 

## 3. Results

**Figure 1:**
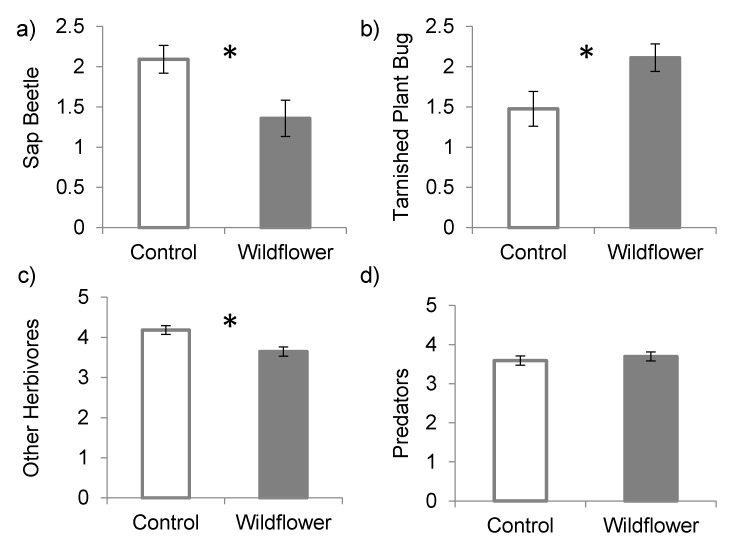
Mean (±SE) abundance of two pests. (**a**) strawberry sap beetle (*Stelidota geminata*); (**b**) tarnished plant bug (*Lygus lineolaris*); as well as (**c**) other herbivores and (**d**) predators sampled by vacuum from plots with a control or wildflower border.

Vacuum sampling within the strawberry plantings revealed that wildflower strip borders had different, sometimes opposing effects, depending on the pest species and functional group (*functional group x treatment F*_(3,5)_ = 14.79, *p* = 0.006). Strawberry plantings with a wildflower border had fewer *S. geminata* per sample (z-ratio = 2.961, *p* = 0.003; Figure 1a) but a greater number of *L. lineolaris* (z-ratio = −2.677, *p* = 0.007; Figure 1b). The number of other herbivores collected in plots with a wildflower border was also lower (z-ratio = 6.525, *p* < 0.0001; Figure 1c) but the number of predators was not different between control plots and plots with a wildflower border (z-ratio = −1.150, *p* = 0.25; Figure 1d). The most abundant herbivore groups included Rhyparochromidae (8.1%), Cicadellidae (6.3%), *L. lineolaris* (4.4%) and *S. geminata* (4.2%). The most abundant predators sampled by vacuuming included Araneae (14.8%), Formicidae (7.3%) and Opiliones (5.1%). 

**Figure 2:**
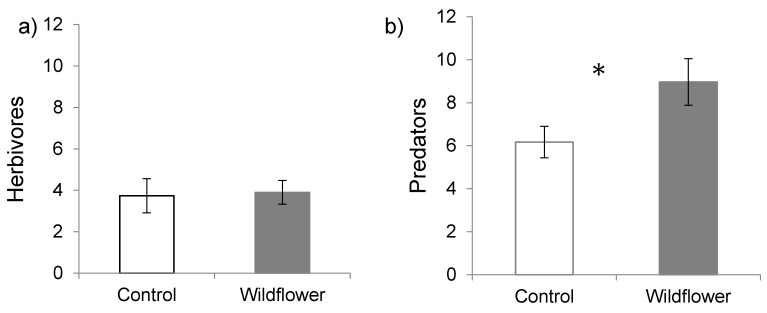
Mean (± SE) abundance of (**a**) herbivores and (**b**) predators sampled from pitfall traps in strawberry plantings with and without a wildflower border.

Pitfall sampling revealed no differences in the abundance of all herbivores in strawberry plantings with a wildflower border compared to controls (*F*_(1,5)_ = 0.07, *p* = 0.79; Figure 2a). However, predator abundances estimated by pitfall traps were greater in plots with a wildflower border (*F*_(1,5)_ = 13.15, *p* = 0.015; Figure 2b). The most abundant herbivores collected in pitfall traps included *S. geminata* (3.9%), Cicadellidae (3.7%) and Aphididae (1%), while the most abundant predators included Araneae (15.9%), Opiliones (6.7%), Formicidae (3.6%) and Carabidae (2.7%).

Although community composition varied strongly by sampling method (*F*_(1,67)_ = 27.26, *p* = 0.001), there was no significant difference in community composition between border treatments (*F*_(1,67)_ = 1.38, *p* = 0.13).

## 4. Discussion

Our findings reveal that wildflower strips have differing effects on pest populations within the crop. As hypothesized, populations of *L. lineolaris*, a generalist feeder, were greater in strawberry plantings with wildflower borders. However, *S. geminata*, which has a narrower feeding niche, was less abundant in plots with a wildflower border in accordance with our predictions. This suggests the possibility that the feeding niche of a pest is a predictor of how wildflower borders will affect the pests’ populations in the crop. For generalists such as *L. lineolaris*, the positive effect of additional food resources may outweigh the negative impacts of increased natural enemies associated with the wildflower borders.

The number of predatory arthropods collected in vacuum samples was not significantly affected by the wildflower border, but pitfall traps revealed greater predator abundances in plots with a wildflower border. These differences likely reflect an increase in ground dwelling predators, most notably carabids, which comprised a greater percentage of the community in pitfall compared to vacuum samples. It is important to point out that in this study only the arthropod populations within the crop were examined; therefore we cannot rule out that natural enemy populations were not greater in the wildflower borders compared to the control borders. Indeed, prior studies have shown that natural enemies inside the wildflower border itself can increase more than the population within the crop [15,16]. These predators in the crop borders may reduce pest migration into the crop. 

Overall populations of herbivores in strawberry plantings with an adjacent wildflower planting were lower than those in control plantings. Lower herbivore numbers may have occurred through top-down effects from the increase in ground-dwelling predators and/or through bottom-up effects on herbivores by decreasing host plant apparency [31,46]. *L. lineolaris* populations increased in strawberry plots with wildflower borders. Similar effects have also been recorded in blueberry plantings with an adjacent wildflower planting of similar composition [16] and in tomato with a diverse wildflower border [35]. We propose that the positive response of *L. lineolaris* to the wildflower border is because of its generalist feeding niche. *L. lineolaris* is likely attracted to and utilizes the wildflowers as a food source and as a bridge to move from surrounding habitats into the strawberry crop. When a generalist is the key pest, the addition of wildflowers may be counter-productive and growers might need to turn to other methods of control. It is important to note however, that the greater number of nymphs observed in plantings with a wildflower border may not result in increases in crop damage. Future studies should explore the potential for flowering strips to impact crop damage or to reduce benefits from other services, such as pollination that may benefit from wildflower strip management. 

Similar to the decrease of herbivores overall, *S. geminata* decreased in strawberry plots with a wildflower border. We suggest that the difference in abundance between treatments for *S. geminata* may be due to increased habitat complexity interfering with host finding behavior and residence time, or through increased predation rates. *S. geminata* feeds on ripe fruits resting on the ground and is therefore potentially susceptible to ground-dwelling predators. *S. geminata* in the strawberry crop may be affected by both bottom-up and top-down factors [30,31]. Because it is likely that both *L. lineolaris* and *S. geminata* could utilize the wildflower plantings as overwintering habitats this suggests that the differential responses to wildflower borders are primarily mediated by differences in feeding preferences or foraging behavior.

## 5. Conclusions

The effects of wildflower borders on pests have important implications for farmers and integrated pest management programs. As more farmers and integrated pest management (IPM) programs implement wildflower borders into their plans for conserving pollinators and natural enemies, they also need to consider how pests are responding. This study suggests that wildflower borders can increase ground-dwelling predators within the crop and decrease the abundance of economically significant pests overall. However, when the main pest can use the resources in the wildflower border, spillover of pests from wildflower plantings may lead to an increase in their population within the crop, and alternative methods of control will be needed to regulate these pests.
